# Dissection of superficial temporal artery for STA-M4 bypass, an integrated method. How I do it

**DOI:** 10.1007/s00701-026-07025-9

**Published:** 2026-09-10

**Authors:** Mohammed O. Alalfi, Sajjad Muhammad

**Affiliations:** 1 Department of Neurosurgery, Medical Faculty and University Hospital Düsseldorf, Heine University Düsseldorf, Moorenstr. 5, 40225 Düsseldorf, Germany; 2 Department of Neurosurgery, Azienda Ospedaliero Universitaria Pisana - AOUP, University of Pisa, Pisa, Italy

**Keywords:** Superficial temporal artery, Middle cerebral artery, Extracranial-intracranial bypass, Moyamoya disease, Dissection technique

## Abstract

**Background:**

Extracranial-intracranial (EC-IC) bypass is an effective revascularization technique for flow augmentation in conditions including Moyamoya disease and ischemic cerebrovascular disorders. Preparation of donor vessel is a key step for EC-IC Bypass surgery. This procedure involves careful dissection and liberation of the Superficial Temporal Artery with subsequent anastomosis to branches of the middle cerebral artery re-establishing flow.

**Methods:**

This article outlines a comprehensive surgical strategy, emphasizing anatomical considerations, procedural nuances, and integration of different schools of dissection with video demonstration making it didactically efficient.

**Conclusion:**

Combined technique for STA dissection is fast, effective and ideal to avoid complications or adverse events.

**Supplementary Information:**

The online version contains supplementary material available at https://doi.org/10.1007/s00701-026-07025-9.

## Introduction

Using Superficial Temporal Artery (STA) to branches of the middle cerebral artery (MCA), Yasargil MG first described the technique for extracranial-intracranial (EC-IC) bypass in 1967 performed then to circumvent an occluded internal carotid artery [10]. Ever since, the technique has evolved particularly in Japan with Kamiyama introducing skeletonization of the STA from the surrounding structures using bipolar forceps [9]. On the other hand the European school maintained the tradition of harvesting the STA with surrounding tissue cuff. In this video, we demonstrate the use of mixed technique using coagulation cutting technique for perforating branches and sharp cutting for connective tissue. The anastomotic part of the STA is skeletonized while the rest of the artery remains intact without skeletonization. This combines the efficiency of the European school by keeping it “simple, efficient while preserving the normal anatomy” and the meticulousness of the Japanese school.

Following the description of the technique by Yasargil and Donaghy at the university of Vermont, the International Cooperative Study of extracranial/intracranial arterial anastomosis (EC-IC Bypass study) was published in 1985 which demonstrated that bypass in addition to medical therapy didn’t prove superior to medical therapy alone in preventing recurrent stroke [1]. Consequently, after this study, the number of bypass procedures performed worldwide plummeted particularly for ischemic stroke being performed in select patients namely patients with stroke with refractory medical therapy and patients with compromised reserve capacity. On the other hand the stance of STA-MCA bypass for moyamoya disease (MMD) remained largely unchanged being the first line therapy given the aggressive natural history of the disease together with inefficacy of medical treatment [6]. Direct STA-MCA bypass in pediatric MMD patients requires careful dissection and special care to fragile vessels during the anastomosis. In this “How I do it” video we demonstrate STA-MCA bypass in a 12 years old patient combining the techniques of two different schools.

## Relevant surgical anatomy

The STA constitutes one of the main terminal branches of the external carotid artery (ECA). The STA runs within the temporoparietal fascia (TPF) which in turn is continuous with the galea cranially. The TPF is considered to be immediately deep to the subcutaneous tissue and as such care should be taken when performing the incision as to not injure the STA given its superficiality. Multiple authors described notable variations in the anatomy of the STA among cadavers necessitating studying of the course of the STA using doppler and palpation methods prior to incision [4, 5, 8]. In its “classical” description, the STA, following its branching from ECA, courses superiorly between the tragus and the temporo-mandibular joint, within the TPF, eventually bifurcating into its anterior (frontal) and posterior (parietal) branches approximately 3—5 cm above the zygomatic arch. The posterior branch of the STA courses superiorly paralleling the superior temporal line eventually anastomosing with the contralateral parietal branch across the midline, posterior auricular artery and occipital artery branches ensuring collateral circulation for adequate perfusion. The posterior branch is the preferred graft for MCA bypass. This is due the fact that the parietal branch aligns better with the MCA recipients keeping the skin incision small and enabling tension free anastomosis. Furthermore the parietal branch is present in the vast majority of individuals with prevalence rates reported being around 96% [3]. The main diameter of the parietal branch ranges from approximately 1.2 mm to 1.7 mm (10). Similar to the STA, the frontotemporal branch of the facial nerve, by many accounts, runs in the same plane as the STA (TPF) although anteroinferior to the anterior branch of the STA in most cases. Some other authors note that the frontotemporal branches of the facial nerve run deeper to the TPF particularly in the loose alveolar connective tissue (LAT). However given that the frontotemporal branch of the facial nerve runs mostly anteroinferior to the anterior (frontal) branch of the STA, it is not of utmost concern to the operating surgeon given that the incision and subsequent craniotomy is done much more posteriorly over the course of the posterior (parietal) branch of the STA.

## Description of the surgical technique

### Pre-operative planning

Preoperative preparation begins with high-resolution vascular imaging, such as digital subtraction angiography (DSA), computed tomography angiography (CTA), or magnetic resonance angiography (MRA) and perfusion scans to map the vascular anatomy, quantify reserve capacity and assess collateral circulation. A Doppler ultrasound is used to identify the course of the STA branches. During anesthesia, normocapnia, normothermia, and stable hemodynamics are meticulously maintained to reduce intraoperative complications as described previously [7].

### Surgical steps

**Fig. 1:**
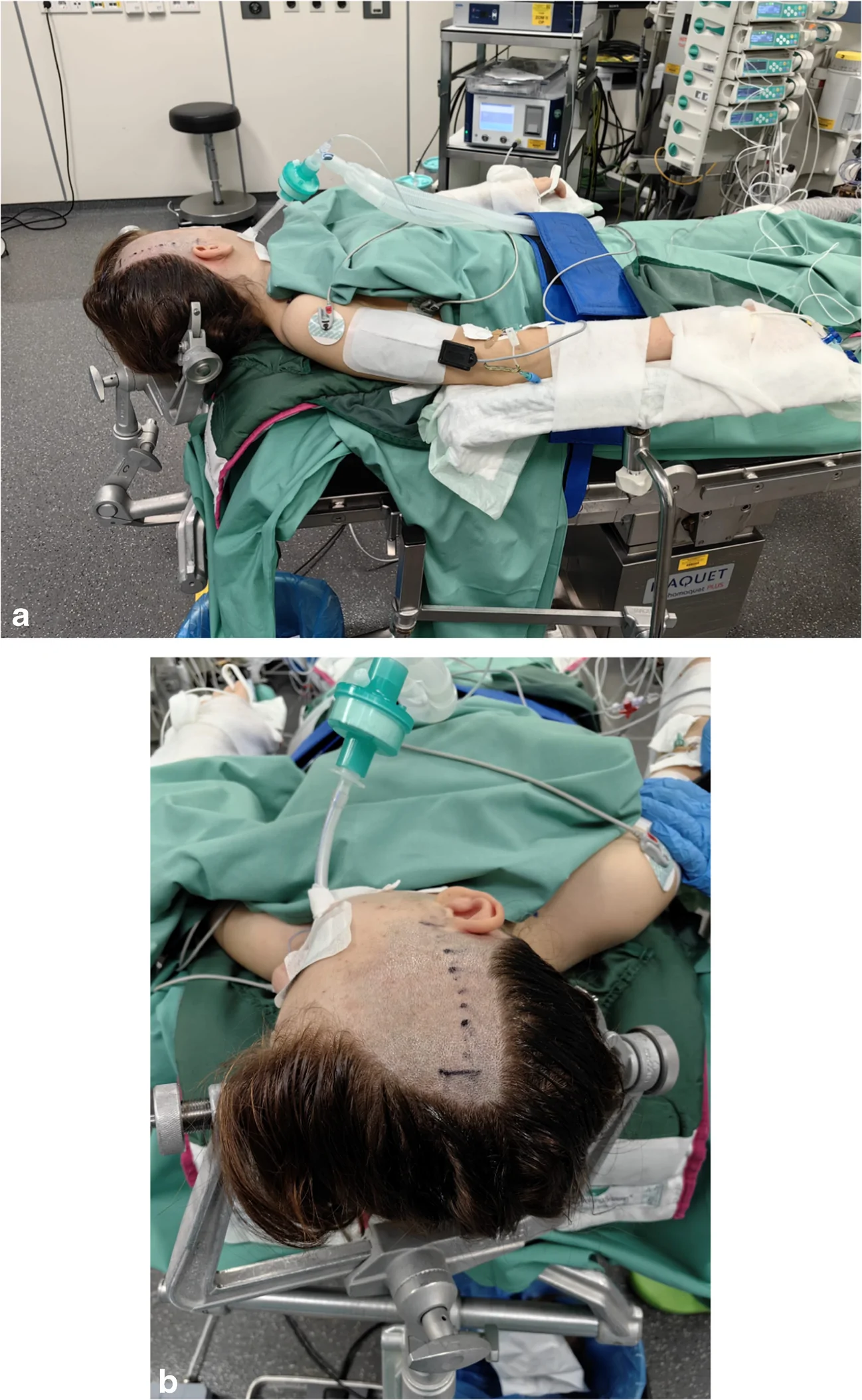
**A** Patient is placed in standard supine position with the head rotated approximately 90 degrees within the Mayfield clamp contralateral to the side of the anastomosis. **B** Incision site is marked over the posterior branch of the STA

The patient is positioned in the standard supine position with the head rotated approximately 90 degrees contralateral to the side of the intended anastomosis and fixed in position using Mayfield clamp. This is demonstrated in Fig. 1 A and B.

The identification of the STA course is a prerequisite for a successful and efficient harvest especially given the variability of the course of the STA and its subdivision point from one specimen to another. Therefore the use of preoperative imaging, handheld doppler ultrasound and palpation techniques are essential steps for identifying the course of the STA prior to incision. This is demonstrated in Fig. 1B where the incision is marked over the posterior branch of the STA which in turn is located with the guidance of doppler ultrasound. Some authors identify the STA prior to induction of the anesthesia given that anesthetics can diminish the pulse to the STA rendering its palpation less sensitive [2].

**Fig. 2:**
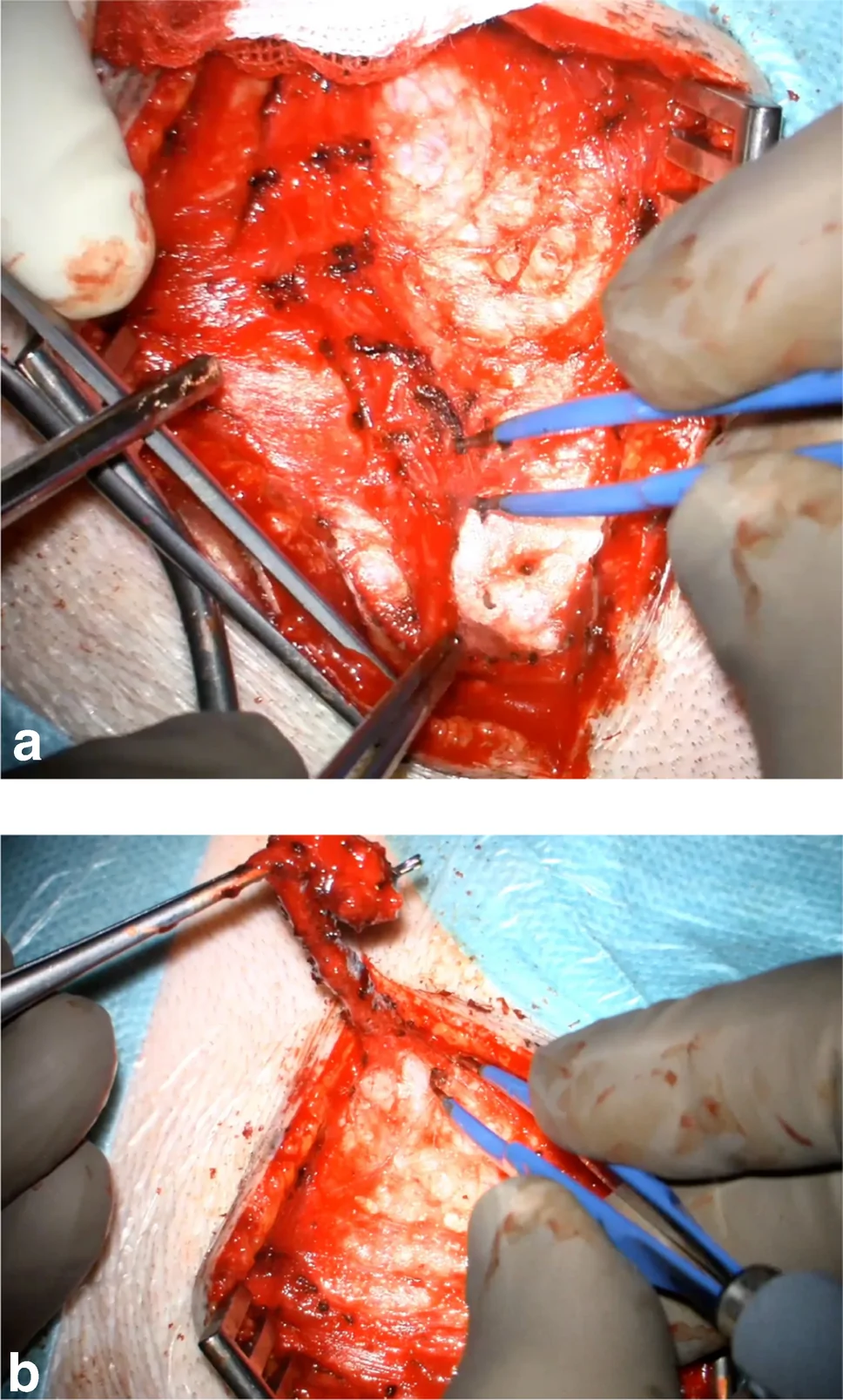

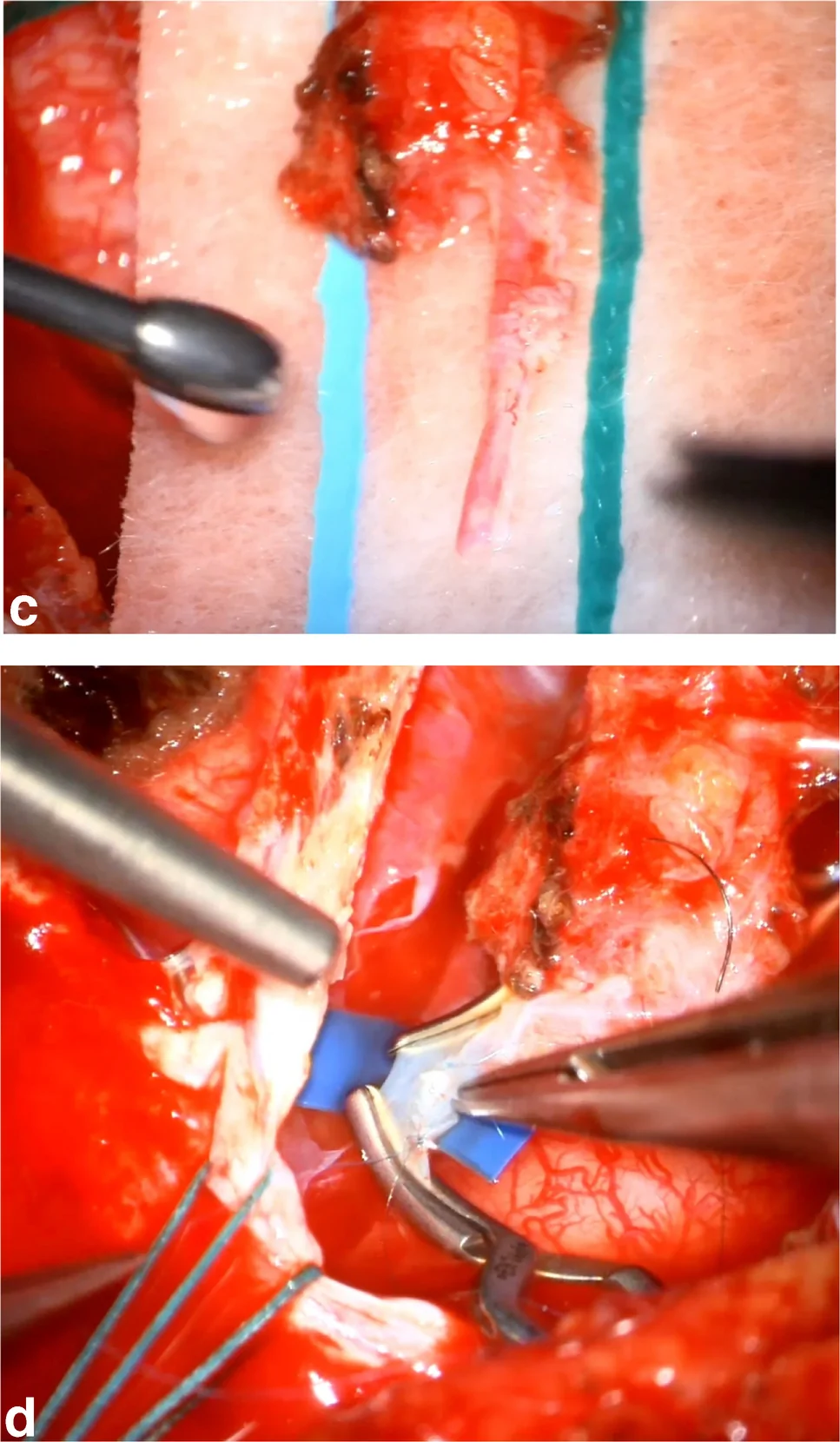
**A** and **B** Intraoperative views demonstrating the seperation of the STA from the surrounding connective tissue cuff using bipolar forceps. **C** Intraoperative view demonstrating the skeletonization of only the distal part of the STA used in anastomosis. **D** Intraoperative view demonstrating STA-MCA (M4) end to side anastomosis

Firstly, a skin incision occurs along the line of the parietal branch of the STA. The skin incision can be initiated either superiorly or inferiorly depending on the surgeon's preference. The STA is then identified and separated from surrounding connective tissue as demonstrated in Fig. 2A and B. The distal end of the STA is then cut and temporarily clipped while also being kept moist with nimodipine solution throughout the entire procedure. Following STA graft preparation, a Y shaped incision is made through the temporal fascia exposing the underlying temporalis muscle which is incised and retracted anteriorly exposing the underlying pterion which in turn is overlying the sylvian fissure in most cases. A mini craniotomy is then performed with dimensions of approximately 4 × 4 cm. Next the underlying dura is opened, subarachnoid dissection is performed and the M4 recipient artery is identified. Henceforth, some surgeons skeletonize the STA from the surrounding connective tissue cuff completely while others do not. In this video we demonstrate the skeletonization of the anastomotic part of the STA from the surrounding tissue cuff while maintaining the connective tissue in the remainder of the STA. The dissection takes place under the microscope and eventually the STA is trimmed obliquely to match the recipient’s vessel. Temporary clips are then placed in the M4 recipient branches proximal and distal to site of anastomosis. Eventually an end-to-side STA-MCA branch anastomosis is then performed using 10–0 monofilament sutures (Fig. 2D). An autologous muscle stamp is placed over the anastomosis to promote secondary hemostasis and prevent anastomotic leakage. The patency is confirmed intraoperatively with doppler flowmetry and indocyanine green angiography Following the bypass, the senior author uses INVOS, a near-infrared spectroscopy (NIRS) system, to ascertain adequate blood flow ipsilateral and contralateral to the bypass. Intraoperatively, 100 mg of aspirin is administered to the patient.

## Indications

This integrated approach is indicated in adult and pediatric patients with symptomatic Moyamoya disease, recurrent chronic ischemia with compromised reserve capacity selected for STA-MCA bypass and stroke refractory to medical therapy.

## How to avoid complications

Meticulous microsurgical technique and intraoperative confirmation of flow using doppler can combat anastomotic failure. Alternatively incising the STA is a possibility particularly for surgeons with less experience for which starting the incision distally could combat this issue. Intraoperative perfusion pressure is important throughout surgery to avoid intraoperative ischemia. On the long run, hyper perfusion syndrome is managed through monitoring of clinical symptoms together with blood pressure reduction.

## Key points summary

1. STA-MCA bypass for pediatric and adult Moyamoya disease remains the first line treatment choice
2. Studying the STA preoperatively is essential using imaging techniques
3. Skeletonization of the STA allows for precise suturing at the cost of stripping the vasavasorum
4. Not skeletonizing the STA is efficient but at the cost of anastomotic bulk that may obscure the luminal edges
5. Combining both techniques leads to more efficient and more precise anastomosis than either alone
6. Integrating encephaloduromyosynangiosis enhances outcome
7. Follow up imaging is important to assess revascularization and avoid complications
8. Strict blood management surveillance is important post operatively
9. Patient education on expected recovery aids compliance
10. This strategy is tailored for progressive ischemic conditions in pediatric and adult patients.

## Supplementary Information

Below is the link to the electronic supplementary material.

ESM 1 Supplementary Material 1 (MOV 417 MB)

ESM 2 Supplementary Material 2 (MOV 417 MB)

## Data Availability

No datasets were generated or analysed during the current study.
